# Experimental modeling and multi-objective optimization of Micro Arc Oxidation process parameters of aluminium alloy joints

**DOI:** 10.1038/s41598-026-51788-1

**Published:** 2026-05-21

**Authors:** Addanki Ramaswamy, M. Bakkiyaraj, Bellamkonda Prasanna Nagasai, S. Malarvizhi, V. Balasubramanian, Robert Cep, B. Swarna

**Affiliations:** 1 Department of Mechanical Engineering, SASI Institute of Technology and Engineering, Tadepalligudem, 534101 India; 2 Department of Mechanical Engineering, Rajalakshmi Institute of Technology, Chennai, 600124 India; 3 Centre for Sustainable Materials and Surface Metamorphosis (CSMSM), Department of Mechanical Engineering, Chennai Institute of Technology, Kundrathur, Chennai, Tamilnadu 600069 India; 4 Centre for Materials Joining and Research (CEMAJOR), Department of Manufacturing Engineering, Annamalai University, Annamalai Nagar, 608 002 India; 5 Department of Machining, Assembly and Engineering Metrology, Faculty of Mechanical Engineering, VSB-Technical University of Ostrava, Ostrava, 70800 Czech Republic; 6 Department of Biosciences, Saveetha School of Engineering. Saveetha Institute of Medical and Technical Sciences, Chennai, 602105 India; 7 University Centre for Research & Development, Chandigarh University, Mohali, 140413 India

**Keywords:** Aluminium alloy, Micro arc oxidation, Optimization, Current density, Inter electrode distance, Oxidation time, Engineering, Materials science

## Abstract

Micro Arc Oxidation (MAO) is an advanced electrochemical surface-processing technique capable of producing hard, dense and adherent ceramic coatings on aluminium alloys, exhibiting superior wear and corrosion properties than traditional anodizing. This research project produced MAO coating on CMT-welded AA6082-T6 aluminium alloy joints and the effect of current density, the oxidation time and the distance in between the electrodes on the coating characteristics were systematically studied using Response Surface Methodology (RSM) based parametric mathematical modelling (PMM). The PMMs generated very accurate predictions of the porosity and hardness of the aluminium alloy joints with an error margin less than 2% and 99% confidence level. Detailed characterization was done to test model predictions. The analysis of the SEM showed how the crater-type discharge channels, micropores, and the patterns of molten-oxide resolidification changed, and optimal parameters resulted in homogeneous and compact structures. The thickness variation between about 58 μm to 110 μm with current density was established by cross-sectional SEM. The XRD patterns showed that, γ-Al_2_O_3_, θ-Al_2_O_3_ and α- Al_2_O_3_ phases existed and were transformed with α- Al_2_O_3_ enrichment at moderate current density (0.19 A/cm^2^) that increased peak hardness. High density of current (0.25 A/cm^2^) inhibited the formation of α- Al_2_O_3_ and high porosity. Optimized MAO conditions of 0.19 A/cm^2^ current density, 20 min oxidation time, and 6-cm spacing between electrodes gave minimum porosity (2.07 vol) and maximum hardness (1459.36 HV). The coatings also exhibited positive wear behaviour, and decreased friction coefficient was observed due to high density of ceramic phase. The overall results of modelling and characterization offer a solid system of application of MAO coating on welded aluminium structures in the high-performance engineering applications.

## Introduction

In the last few decades, hard anodization (HA) has received a lot of attention as a relatively low-cost technique to enhance the corrosion resistance of aluminum alloys through the formation of a thick, amorphous, and porous oxide layer that is usually more than 25 μm^1,2^. Nevertheless, despite its extensive application, HA can be increasingly considered less sustainable due to using electrolytes with high concentrations of strong acids, large current densities, and the necessity to operate at low temperatures, which makes the process energy-intensive and unfriendly to the environment^3,4^. The further development of the aerospace applications exposes aluminum components to even stricter service conditions, which stimulates the search of cleaner and more efficient substitutes of the classical HA. In this context, MAO has developed as an exceptional surface modification method for various non-ferrous alloys^5–8^. MAO is valued for its ability to form hard coatings with improved resistance towards corrosion, wear as well as strong coating–substrate adhesion^9,10^. Unlike HA, MAO operates under markedly different electrolyte conditions and voltage regimes, and the mechanism by which the coating forms is fundamentally distinct^11^.

MAO is a pioneering surface modification approach used to produce hard, uniform and dense ceramic coatings. In the MAO coating process, the substrate acts as an anode submerged in an aqueous solution. Asymmetrically alternating current voltages are applied between the anode and cathode. The voltage range for anodic phase is maintained between 150 and 1000 V, while in the cathodic phase, it varies from 0 to 100 V^12^. MAO coating process is generally characterized in three levels of stages. In the first level, with the increase in time, the voltage increases and sparks are generated on the surface but a thin oxide layer (Al₂O₃) forms. The oxygen gas that adsorbs on the oxide film results from the oxidation of hydroxyl and water ions. It leads to a four‑phase complex system called substrate/dielectric oxide/oxygen gas/electrolyte. Dielectric oxide film and oxygen gas envelope electrical conductivities are very low, and the cell voltage finally increases. In the second level, the dielectric film breaks down and small white sparks, which can be observed move over the specimen’s surface^13^. The voltage increases further during this stage. During the third level, uniform sparking could be generated on the surface of the metal and finally voltage becomes stable because of the steady discharge and uniform growth rate of the film. The density of the sparks decreases by the gradual rise in the spark size. Usually, two inflection points occur during the process. The first inflection point matches with the breakdown voltage beyond which sparking occurs, and the time taken to breakdown voltage is known as the ignition time. The second inflection point, known as the critical voltage point, is where the spark changes and attains stability^14^.

When the generated electron temperature rises to 104 K, the material in the channel is heated by the intense electric field and the anionic components enter the channel. The aluminium and its alloying substituents melt from the substrate and get oxidized by entering the channel due to the high temperature. Then these oxidized aluminium species are ejected from the channel to the coating surface in contact with the electrolyte, resulting in an increase of coating thickness. Finally, the products are deposited on the walls as the discharge channels cool^11,15^. This process continues over several distinct locations on the coating surface, which results in the formation of a thin layer. Several studies have examined the corrosion behaviour of MAO coated alloys, and their results displayed that the MAO process enhances the corrosion resistance properties of the underlayer at a significant level^16–18^.

This work presents a focused investigation into the optimization of micro-arc oxidation (MAO) coatings applied to Cold Metal Transfer (CMT) welded AA6082-T6 aluminium joints, with particular attention to the weld metal region. While earlier studies on MAO have largely concentrated on coating formation mechanisms and corrosion behaviour of base alloys, there has been limited effort devoted to systematic parameter optimization for welded components^3,19^. By targeting the weld zone and developing a structured parametric approach, this study addresses an important gap in the existing literature on surface modification of CMT welded aluminium structures.

## Experimental work

**Fig. 1:**
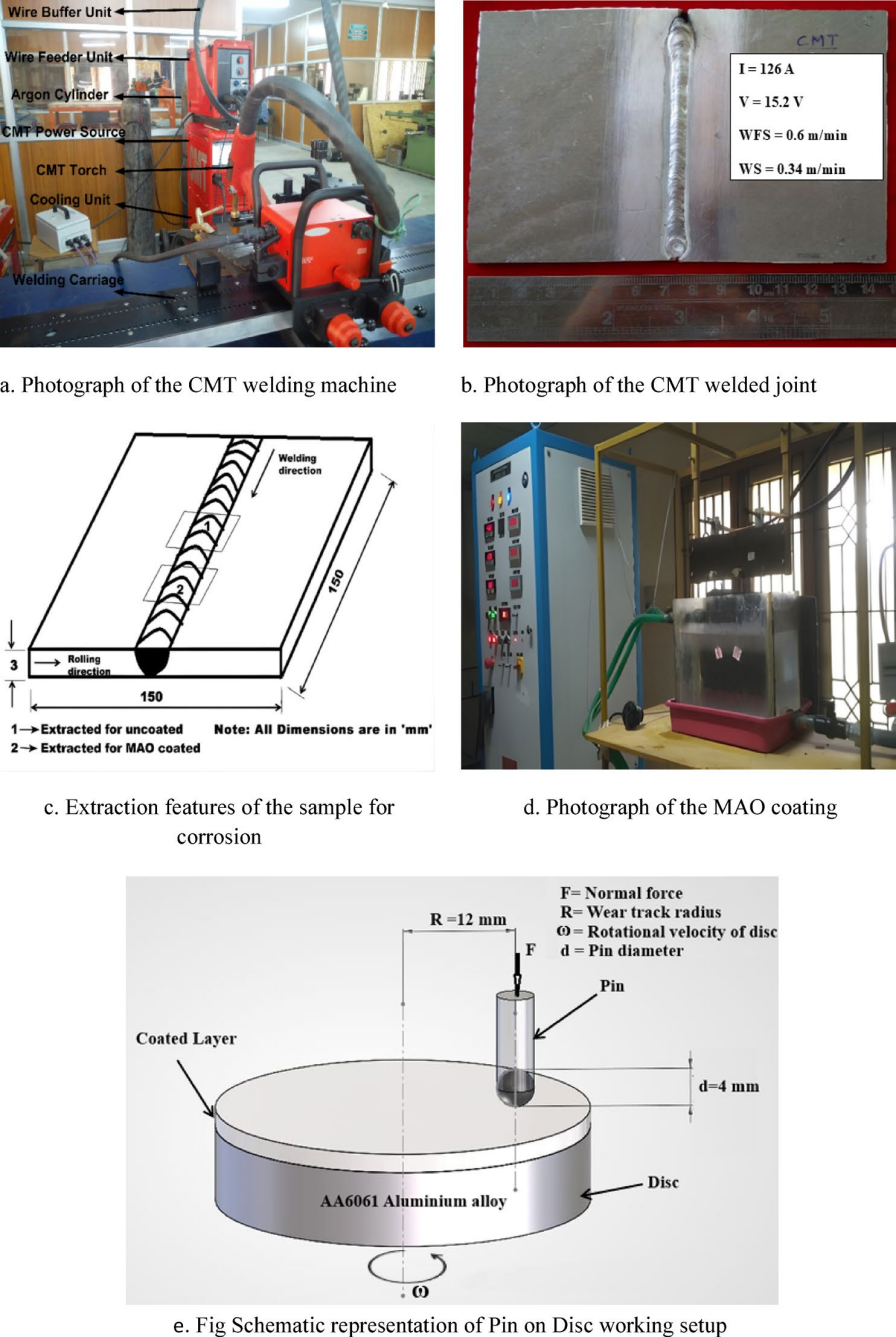
Experimental works and specimens.

In this work, AA6082 aluminum alloy (3 mm thickness) served as the material while ER4043 filler wire of 1.2 mm diameter was used for experimentation. Its nominal composition is specified in Table 1. The values listed in Table 1 are obtained from the Energy-dispersive X-ray spectroscopy. Optimized welding parameters utilized to manufacture the joint is shown in Table 2. These sheets were divided into sections that were 150 mm long and 75 mm wide, while maintaining the 60 ° groove angle that was included between the sheets. Pure argon was employed as shielding gas flowing at 15 L per minute. These sheets were wire brushed to remove the oxide layer. CMT advanced 4000R machine (Fig. 1a) was used to join these sheets in a single pass with direct current electrode positive (DCEP) polarity. Close up view of the CMT welded specimens is shown in Figure. 1.b. The corrosion specimens sectioned from the weldment (Fig. 1.c) cover the parent material, heat affected zone (HAZ) and fusion zones. The specimens were machined to a length and width of 20 mm, thickness as 3 mm. Then they are polished step-by-step using abrasive papers of 600 grit, 1200 grit, and 2000 grit. After polishing, specimens were dressed in an ultrasonic acetone bath, followed by an ethanol rinse and drying, before undergoing MAO (Fig. 1d). The MAO process was employed utilizing a voltage range from 0 to 600 V, current up to 600 A, and pulse frequencies between 50 and 5000 Hz, with the duty cycle adjustable from 10% to 90%. The electrolytic solution chosen was a mixture of sodium meta-silicate (Na_2_SiO_3_·9H_2_O) of 2 g and potassium hydroxide (KOH) of 4 g for one liter of distilled water giving a total concentration of approximately 6 g/L solids and a measured pH of about 12.5 at 25 °C using a calibrated pH meter. The electrolyte conductivity was maintained in the range of 12–15 mS/cm at room temperature, and the solution was renewed after every five experimental runs to minimize potential depletion of silicate and potassium species. The surface characteristics of the resulting coatings were analysed using a JSM-5600LV scanning electron microscopy (SEM). MAO coated surface was assessed using a Vickers microhardness tester to measure the microhardness in accordance with ASTM E384. A force of 50 gf was applied for a duration of 15 s, which is appropriate for relatively thin coatings in micron-meter range. The surface irregularities (R_a_) were assessed using a contact stylus profilometer (such as a Mitutoyo SJ-210) following ISO 4287 standards. A cutoff length of 0.8 mm was used, and measurements were taken over four to five traverses to ensure repeatability. Porosity (vol %) was determined through image analysis of cross-sectional optical and SEM micrographs using ImageJ software with appropriate thresholding techniques. The analysis followed the metallographic guidelines outlined in ASTM E2109. ASTM G99 standard was employed for performing the Pin on Disc wear test (Fig. 1e). Pin on Disc apparatus (DUCOM TR-20LE) was utilized to evaluate wear characteristics under a 330 g normal load, with a 15 Hz frequency, using a steel ball with a diameter of 4 mm with a track velocity of about 0.94 m/s, and a test duration of 5 min, giving a total sliding distance of nearly 300 m. The experiments were conducted under dry conditions at ambient temperature using a 4 mm diameter EN31 steel ball as the counterface. The BRUKER D8 X-ray diffraction (XRD) technique utilizing a standard 1.54 Å Cu Kα radiation at 40 kV and 30 mA, was utilized to examine the phases in ceramic coatings in accordance with ASTM E1426 standard. The analysis was conducted with a step increment of 0.02°, scanning velocity of 2°/min, and a phase range spanning from 20° to 100°.

**Table 1:** Elemental composition (wt%) of base material and filler metal.

	Si	Fe	Cr	Zn	Mg	Ti	Cu	Mn	Al
AA6082-T6	0.76	0.27	-	0.031	0.97	0.022	0.09	0.42	Bal.
ER4043	5.4	0.7	0.06	0.11	0.06	0.019	0.4	0.06	Bal.

**Table 2:** Welding variables.

Mode	Welding current (A)	Wire feed speed (mm/min)	Arc voltage (V)	Torch Travel speed (mm/min)	Heat input (kJ/mm)
CMT-MIG	126	6000	15.2	340	0.271

## Results

### Performing the trials and recording the response

**Fig. 2:**
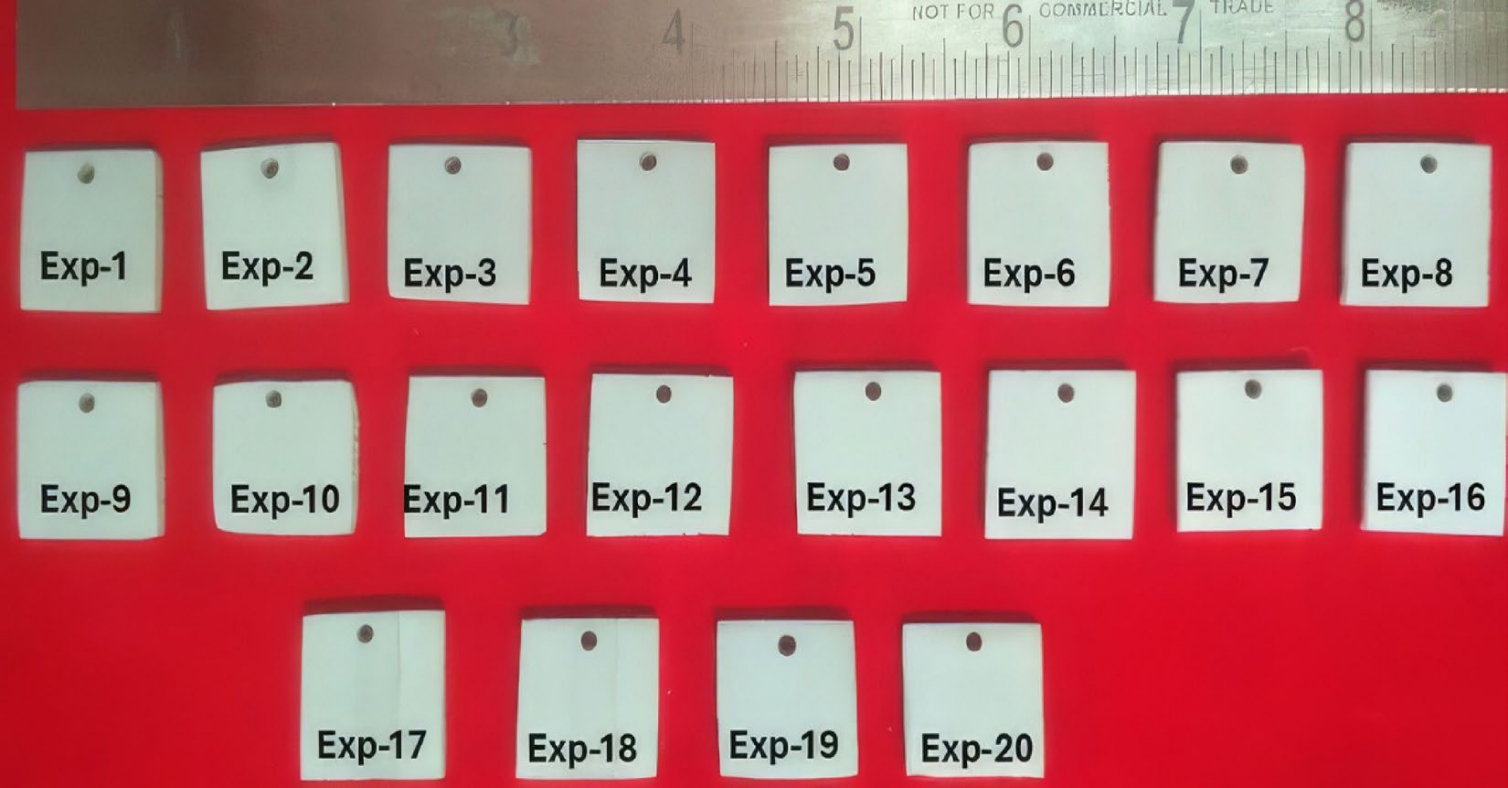
Photograph of the MAO coated specimens.

The optimized parametric range of the MAO process was found, and it is presented in Table 3 with detailed observations. The process parameters with their levels are shown in Table 4. In order to reduce the number of trials, a 3 factors, 5 levels central composite design matrix was used in this investigation and the developed design matrix with 20 experimental conditions sets are shown in Table 5. Figure 2 displays the image of the specimens with coatings. The first 8 runs (or conditions) in the design matrix represent the factorial points and the next 6 runs (or conditions) in the design matrix represent the star points or corner points. The last 6 runs (or conditions) in the design matrix represent the centre points. All the three factors are placed at the middle level, and the experimental conditions are repeated 6 times to check the repeatability of experimental results. The experimental conditions were done as prescribed in the design matrix and the responses of each test were recorded and tabulated. The suitability of the established empirical relationship was evaluated using the ANOVA method, facilitated by the Design Expert software.

**Table 3:** Finding the levels of the MAO process parameters.

Condition	SEM Micrograph	Observations
If I < 0.12 A/cm^2^		Coating surface contains only γ-Al_2_O_3_ with little amount of α-Al_2_O_3_ (Non uniform surface roughness)
If I > 0.25 A/cm^2^		wobbly porous oxide layer exists due to the over-usage of current
4 cm < D < 8 cm	**-**	Fixed constraint
If T < 10 min		Micro pores and voids occurs in the microstructure
If T > 30 min		Discharge channel, craters and cracks were formed

**Table 4:** Levels of used MAO parameters.

Parameters	Symbol	Unit	Levels				
			−1.68	−1	0	+ 1	+ 1.68
Current density	I	A/cm^2^	0.12	0.15	0.19	0.22	0.25
Oxidation time	T	min	10	14.05	20	25.94	30
Inter-electrode distance	D	cm	4	4.81	6	7.19	8

**Table 5:** Design matrix.

Trial number	Factors			Responses	
	I	D	T	Coating porosity (Vol.%)	Coating hardness (HV)
1	0.15	4.81	14.00	2.6	1062
2	0.22	4.81	14.00	4.22	1309
3	0.15	7.19	14.00	2.96	1081
4	0.22	7.19	14.00	4.97	1301
5	0.15	4.81	26.00	2.01	1225
6	0.22	4.81	26.00	3.2	1403
7	0.15	7.19	26.00	2.23	1226
8	0.22	7.19	26.00	3.88	1369
9	0.13	6.00	20.00	2.16	1141
10	0.24	6.00	20.00	4.76	1473
11	0.19	4.00	20.00	2.02	1350
12	0.19	8.00	20.00	2.96	1343
13	0.19	6.00	9.91	3.88	1032
14	0.19	6.00	30.09	2.32	1226
15	0.19	6.00	20.00	2.37	1460
16	0.19	6.00	20.00	2.4	1465
17	0.19	6.00	20.00	2.24	1463
18	0.19	6.00	20.00	2.27	1461
19	0.19	6.00	20.00	2.22	1464
20	0.19	6.00	20.00	2.36	1462

### Determination of parameter limits from preliminary trials

The major influencing parameters like current density (I), oxidation time (T) and inter electrode distance (D) were selected. Several trials were performed on the specimen obtained from the weld metal zone of the CMT welded AA6082 aluminium alloy joint to identify the minimum and maximum limits of the MAO parameters. The following are the inferences that were made while fixing the limits of the MAO process parameters by trial experiments.

I. If I value was less than 0.12 A/cm^2^, then the micro arc generated was not sufficient and due to this, the α- Al_2_O_3_ content formed towards the substrate interface was less and the coating surface contains only γ- Al_2_O_3_ with little amount of α- Al_2_O_3_.The resulting coatings exhibited hardness values under 800 HV, porosity greater than 8 vol%, that leads to the incomplete development of the α-Al₂O₃ phase.
II. If I value was greater than 0.25 A/cm^2^, then the coated substrate consists of wobbly and porous oxide film on the surface due to the extra usage of the current. The resulting coatings produced thicker coatings (approximately 101 μm) but also led to porosity above 12 vol% and pronounced microcracking.
III. The electrode holder’s fixed constraint required the D value to be set between 4 and 8 cm. For inter-electrode distance, values outside the 4–8 cm range caused unstable discharge behavior and significant local thickness variations. Within 4–8 cm, however, porosity fluctuated by only ± 1 vol% and hardness by approximately ± 50 HV, indicating stable and reproducible coating formation.
IV. Micropores and voids form in the microstructure of the coated surface by maintaining an T value of less than 10 min. and hence the hardness recorded was very low. Shorter durations yielded coatings with hardness below 700 HV and porosity exceeding 10 vol%, indicating insufficient oxide growth.
V. If T value was greater than 30 min, then discharge channel, craters and cracks were formed due to the accumulation of high thermal energy and this thermal energy cannot be dissipated easily by the electrolytic solution because of the porous structure of the coating. Longer durations resulted in visible discharge craters and a reduction in hardness, attributed to thermal degradation.

### Formulation of empirical relationships

The responses like porosity and hardness of the coated surface are a function of MAO coating variables such as I, T as well as D and hence, it can be written as

1 $${\mathrm{Z}}\,=\,{\text{f }}\left( {{\mathrm{I}},{\text{ D}},{\text{ T }}} \right)$$

The polynomial equation for the three chosen parameters and their interaction effect could be written using Design Expert as listed by many researchers^20–23^.

Many experimental procedures are available in the Design-Expert software for finding the regression coefficients^3^. Here in this investigation, a central composite design was chosen which suits the second order response surface. Empirical relationships were formulated with the determined coefficients to estimate the porosity and hardness of the MAO coated aluminium alloy specimens. The empirical relationships given in the coded form are presented below.

2 $$\begin{aligned}{\text{Coating porosity }}\left( {\mathrm{P}} \right){\text{ }}=&{\text{ }}\{ +{\mathrm{17}}.{\mathrm{7}}0{\text{ }} - {\text{ }}\left( {{\mathrm{118}}.{\text{72 }}\left( {\mathrm{I}} \right)} \right)-\left( {{\mathrm{1}}.0{\text{7 }}\left( {\mathrm{D}} \right)} \right)-\left( {0.{\text{32 }}\left( {\mathrm{T}} \right)} \right){\text{ }}+{\text{ }}\left( {{\mathrm{2}}.{\text{55 }}\left( {{\mathrm{ID}}} \right)} \right)\\&-\left( {0.{\text{47 }}\left( {{\mathrm{IT}}} \right)} \right)-({\mathrm{3}}.{\mathrm{67X1}}{0^{ - \,{\mathrm{3}}}}\left( {{\mathrm{DT}}} \right)){\text{ }}+{\text{ }}\left( {{\mathrm{366}}.{\text{22 }}\left( {{{\mathrm{I}}^{\mathrm{2}}}} \right)} \right){\text{ }}+{\text{ }}(\left( {0.0{\text{74 }}\left( {{{\mathrm{D}}^{\mathrm{2}}}} \right)} \right){\text{ }}\\&+{\text{ }}({\mathrm{8}}.{\mathrm{92X1}}{0^{ - \,{\mathrm{3}}}}\left( {{{\mathrm{T}}^{\mathrm{2}}}} \right))\} {\mathbf{Vol}}{\text{ }}\%\end{aligned}$$

3 $$\begin{aligned}{\text{Coating hardness }}\left( {\mathrm{H}} \right){\text{ }}=&{\text{ }}\{ - {\mathrm{3771}}.{\text{44 }}+{\text{ }}\left( {{\mathrm{22378}}.{\mathrm{7}}0{\text{ }}\left( {\mathrm{I}} \right)} \right){\text{ }}+{\text{ }}\left( {{\mathrm{398}}.{\text{14 }}\left( {\mathrm{D}} \right)} \right){\text{ }}+{\text{ }}\left( {{\mathrm{161}}.{\mathrm{8}}0{\text{ }}\left( {\mathrm{T}} \right)} \right)\\&-\left( {{\mathrm{186}}.0{\text{7 }}\left( {{\mathrm{ID}}} \right)} \right)-\left( {{\mathrm{86}}.{\mathrm{9}}0{\text{ }}\left( {{\mathrm{IT}}} \right)} \right)-\left( {0.{\text{77 }}\left( {{\mathrm{DT}}} \right)} \right)-\left( {{\mathrm{45155}}.{\text{29 }}\left( {{{\mathrm{I}}^{\mathrm{2}}}} \right)} \right)\\&-\left( {{\mathrm{29}}.{\text{19 }}\left( {{{\mathrm{D}}^{\mathrm{2}}}} \right)} \right)-\left( {{\mathrm{3}}.{\text{28 }}\left( {{{\mathrm{T}}^{\mathrm{2}}}} \right)} \right)\} {\mathbf{HV}}\end{aligned}$$

### Checking adequacy of formulated relationships

The efficacy of the established empirical relationship was estimated via the ANOVA technique. Table 6 presents the ANOVA results for the responses of porosity and hardness of the coated surface. From the tabulated results, the model F-value for the coating porosity and coating hardness. MAO coated weld metal zone of CMT AA6082 aluminium alloy joints are 109.34 and 11970.88 respectively which signifies that the models are statistically significant. Therefore, the results clearly specify that the model terms D, I, T, ID, D^2^, I^2^ as well as T^2^ are significant for the responses coating porosity and model terms D, I, T, ID, DT, IT, D^2^, I^2^ as well as T^2^ are significant for the responses coating hardness. The factors are insignificant only when the p-value > 0.1 i.e., at 99% confidence level. Model terms are statistically significant when Prob > F value is less than 0.05^19,24^. The responses for coating porosity and coating hardness do not show a significant lack of fit, as indicated by the lack of fit F-values of 4.80 and 1.24, respectively. This recommends that the lack of fit is good. The values of the coefficients are tabulated in the Table 7. The adequate precision values of 32.823 (porosity) and 315.946 (hardness), which are well above the required threshold of 4, indicating a strong signal-to-noise ratio.

**Table 6:** ANOVA test results.

Term	Coating porosity		Coating hardness	
	F-value	*p*-value, Prob > F	F-value	*p*-value, Prob > F
Model	109.34	< 0.0001	11970.88	< 0.0001
I	515.14	< 0.0001	33931.38	< 0.0001
D	56.50	< 0.0001	21.35	0.0009
T	160.57	< 0.0001	11868.63	< 0.0001
ID	5.40	0.0424	122.84	< 0.0001
IT	4.67	0.0560	681.16	< 0.0001
DT	0.33	0.5784	61.87	< 0.0001
I^2^	173.57	< 0.0001	11272.63	< 0.0001
D^2^	9.63	0.0112	6299.18	< 0.0001
T^2^	89.05	< 0.0001	51513.55	< 0.0001
Lack of fit	4.80	0.0550	1.24	0.4112
R-squared	0.9999		0.9999	
Pred R-squared	0.9346		0.9995	
Adeq precision	32.823		315.946	
Significant	Yes		Yes	
Lack of fit	Insignificant		Insignificant	

**Table 7:** Co-efficient and its estimated factors.

Coefficient	Coating porosity (Vol%)	Coating hardness (HV_0.5_)
Intercept	2.30	1462.55
I	0.79	98.58
D	0.26	−2.47
T	−0.44	58.31
ID	0.11	−7.75
IT	−0.099	−18.25
DT	−0.026	−5.50
I^2^	0.45	−55.32
D^2^	0.11	−41.35
T^2^	0.32	−118.25

The adequacy of the developed models was evaluated using R^2^, adjusted R^2^, predicted R^2^, lack of fit, and residual analysis. The R^2^ and adjusted R^2^ values (0.9999) indicate an excellent fit between experimental and predicted responses. More importantly, the predicted R^2^ values were 0.9346 for porosity and 0.9995 for hardness. The difference between predicted and adjusted R^2^ is less than 0.2 confirming good agreement and indicating that the models possess strong and predictive capability rather than overfitting.

The slightly lower predicted R^2^ for porosity may be attributed to inherent experimental validity within the CCD design; however, its value (> 0.90) still confirms reliable prediction. Model adequacy is further supported by high adequate precision (> 4), and randomly distributed residuals within ± 3σ, indicating absence of systematic error. In addition, independent validation experiments showing prediction errors below 2% and cross-validation results (Q^2^ = 0.93–0.94) further confirm the robustness and generalization ability of the developed models.

Even though the experimental dataset is a 20 run (on a Central Composite Design (CCD)) based dataset, the CCD is statistically optimized to create second-order response surface models with a minimal number of experiments. Overall, the statistical indicators confirms that the developed models are both adequate and predictive within the studied parameters range.

### Optimizing MAO coating process parameters

**Fig. 3:**
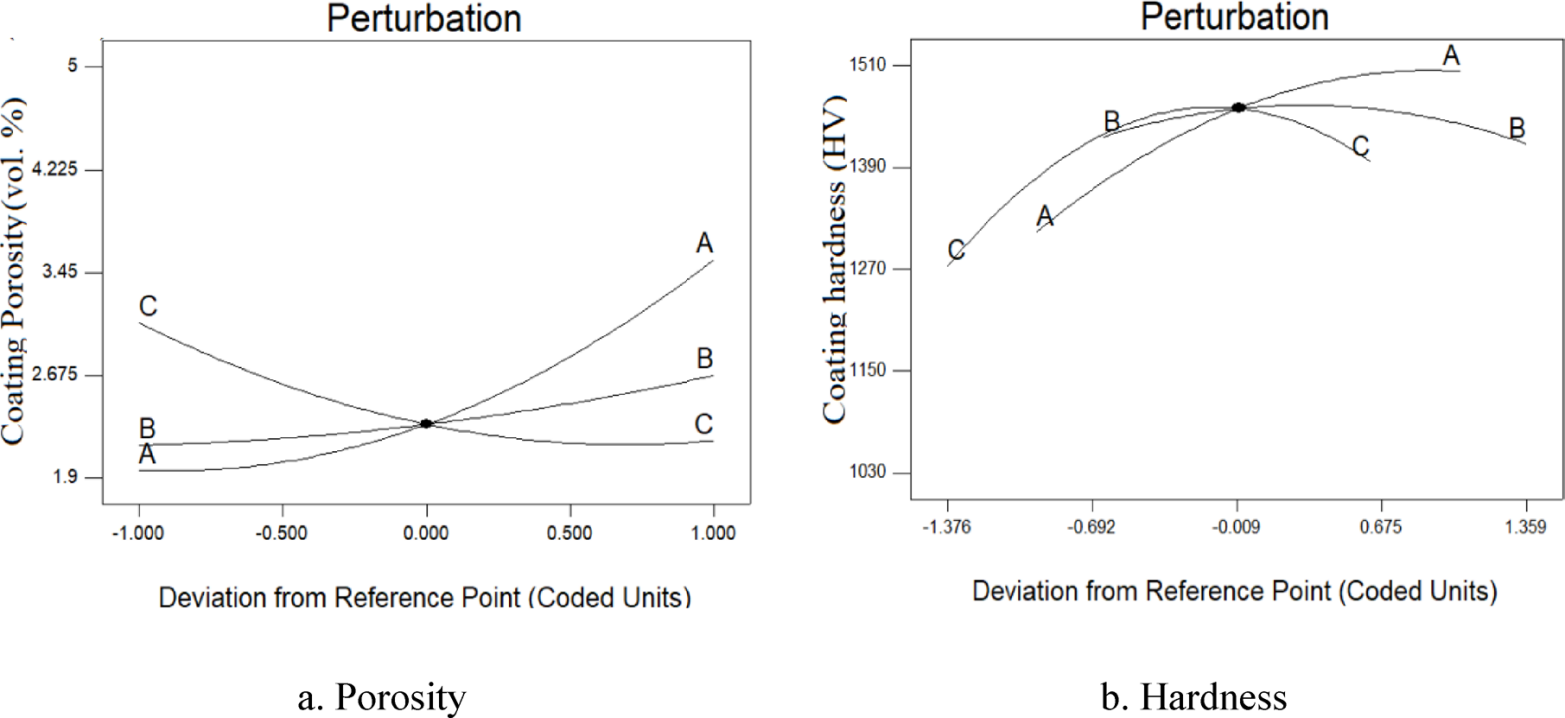
Perturbation graph.

**Fig. 4:**
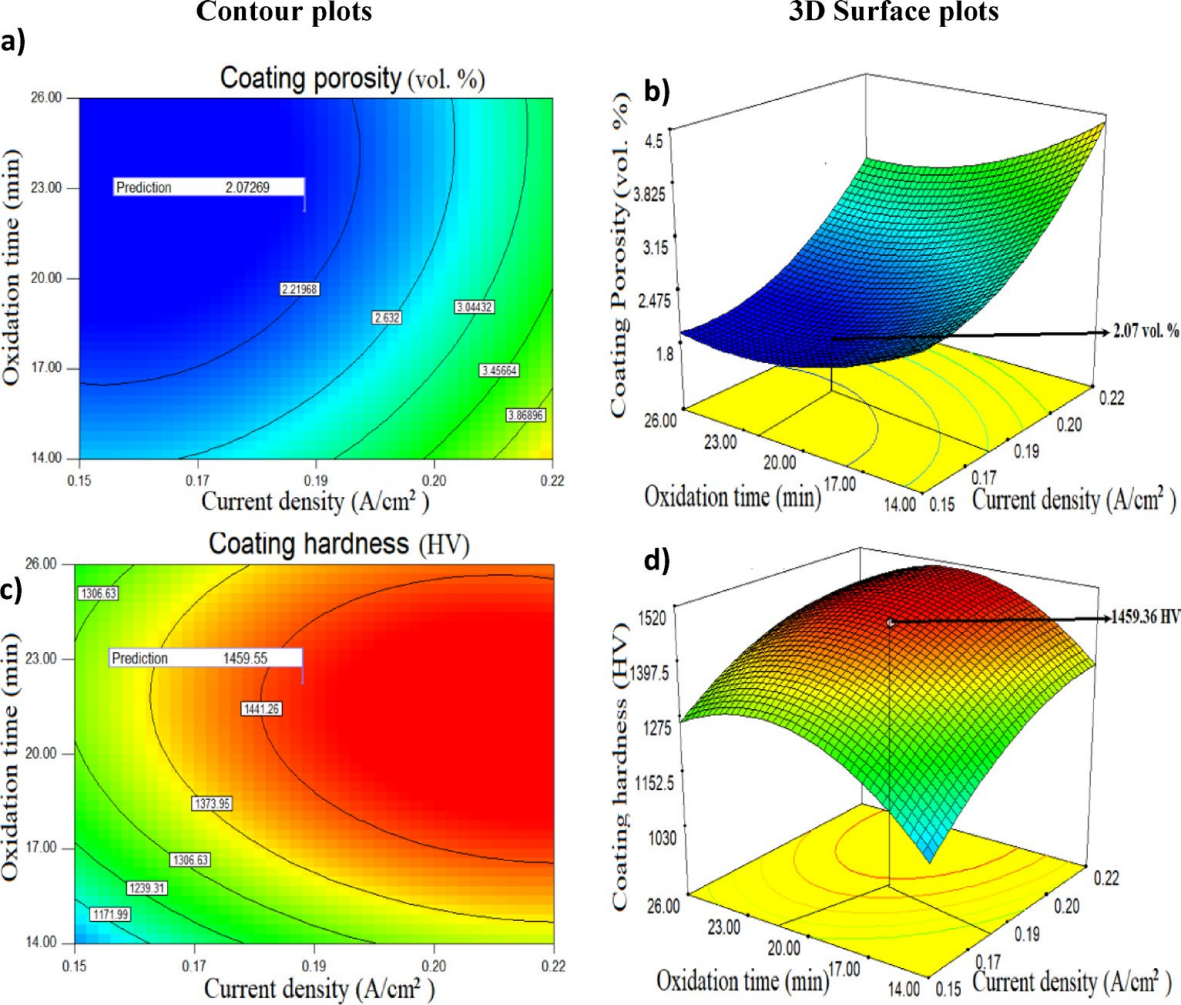
Interaction effect of MAO coating process parameters.

**Fig. 5:**
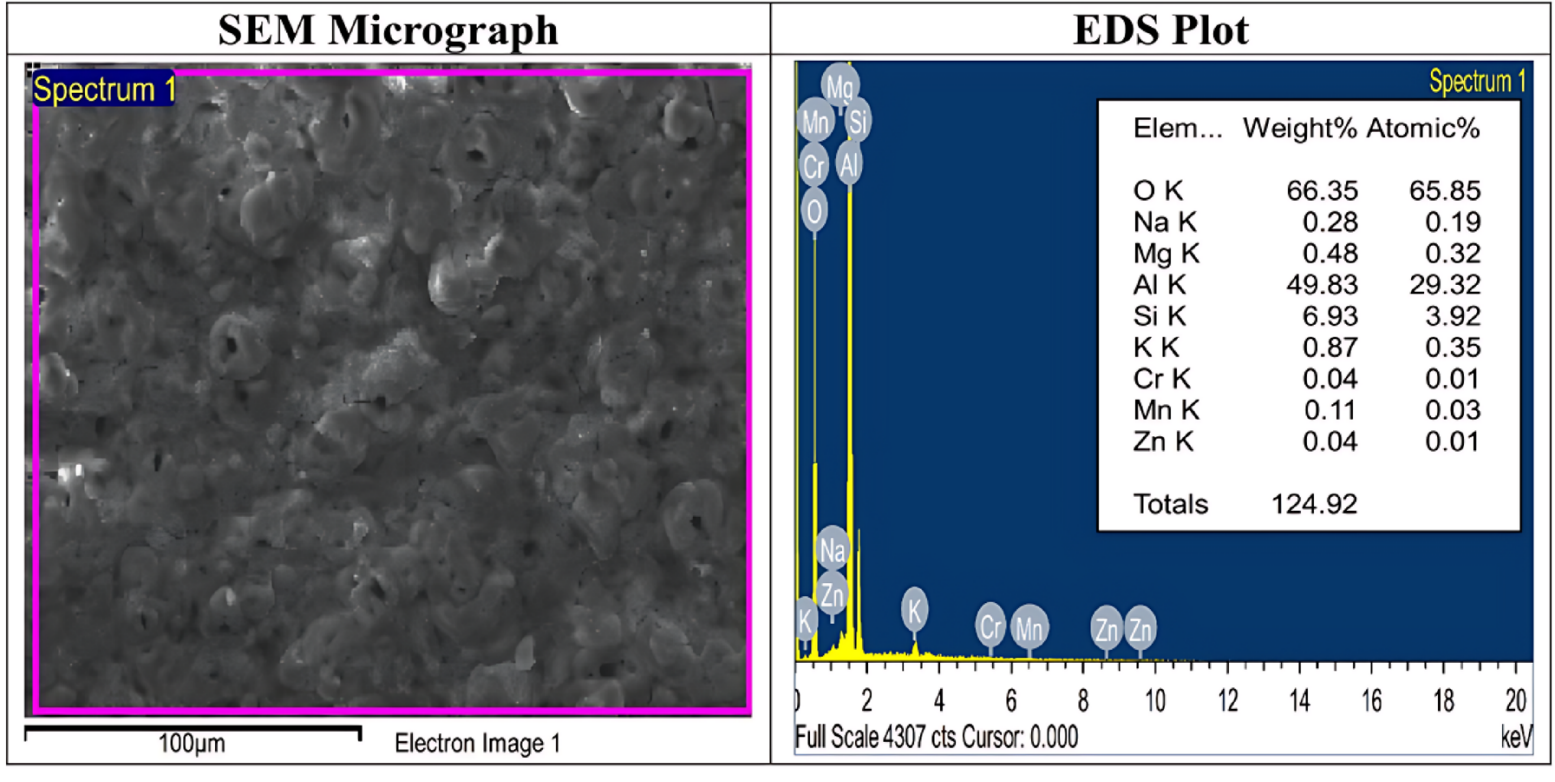
SEM with EDS of the MAO coated surface for the optimized parameter.

In this current investigation, the method chosen was RSM to optimize MAO process parameters. The main objective is to optimize MAO process variables for achieving the coating with less volume percentage of pores and maximum hardness. The perturbation graph of MAO coated aluminium alloy joint for the responses coating porosity and hardness are depicted in Figs. 3a-b respectively. The response surface plot and three-dimensional contour surface plots for the interaction influence of MAO process parameters are displayed in Fig. 4. From the surface graphs, the combined influence of T and I on porosity and hardness of the coated surface could be understood. It is noticed from Fig. 3b that the coating hardness rises with the rise in the I, while the coating hardness rises with the rise in D and T up to a certain point and then starts decreasing. But the effect observed is very less in D. Similarly coating porosity rises with increase in I and D. But the increment in coating porosity is almost negligible with the rise in the D. While the coating porosity decreases with the increase in oxidation time. The bottommost and topmost points in the response plot indicates the minimum and maximum feasible responses i.e., coating porosity and coating hardness respectively. From the contour and surface plot results, it is clearly evident that minimum porosity is noted as 2.07 Vol % and maximum hardness is recorded as 1459.36 HV. The MAO process parameters, as detailed in Table 8, include a I of 0.19 A/cm², T of 28 min with D of 6 cm which result in the lowest porosity and highest hardness. SEM with EDS of the MAO coated surface for the optimised parameter is shown in Fig. 5. It infers that the even distribution of potassium, sodium and silicon elements on the aluminium alloy welded joint. The addition of Si to the oxide layer, promotes the development of mullite and γ-Al₂O₃ phases, which contributes to the characteristic porous and volcanic surface morphology reported in similar systems^27^.

**Table 8:** Optimized parameters.

Parameters	
Current density (A/cm^2^)	0.19
Inter-electrode distance (cm)	6
Oxidation time (min)	20

### Validation of optimization procedures

**Fig. 6:**
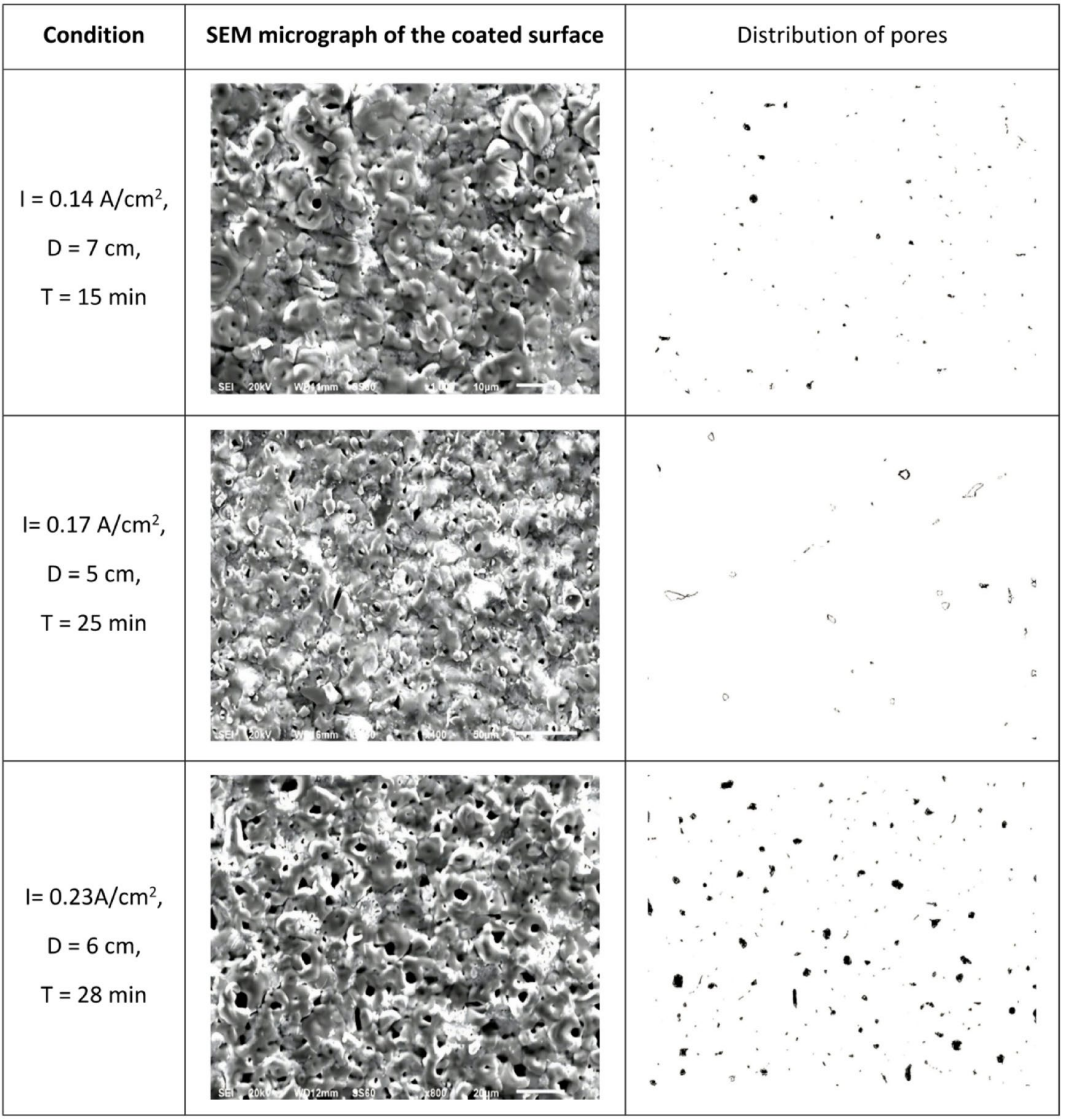
SEM micrograph of the validation test experimental conditions.

A range of MAO process parameters that were randomly selected to validate the empirical relationships are not available in the design matrix. In order to validate the developed empirical correlations, it is essential to find whether the empirical relationships match with the predicted results. Therefore, the results are checked for validation by conducting three more experimental conditions (parametric value in the range) that are not specified in the design matrix. The conditions along with the SEM micrograph of the validated test specimens is evident from Fig. 6. The results of the validation trials are listed in Tables 9 and 10. It clearly indicates that the established relationships are good agreement with a prediction error of less than 2%. The results of the ANOVA test indicate that current density stands out as the most influential factor when compared to the other variables. The validation experiments (Fig. 6) were done with the use of a new combination of MAO parameters not available in the original RSM design matrix, but within the set factor levels. The SEM micrographs and pore distribution maps have the characteristics of the coating, which proceeds in accordance with the RSM predictive, with controlled discharge characteristics and decreased porosity^28^.

**Table 9:** Validation test results for the response coating porosity.

Exp. No	Current density (A/cm^2^)	Oxidation time (min)	Inter electrode distance (cm)	Coating porosity (Vol %)		Error (%)
				Actual	Predicted	
1	0.14	15	7	2.66	2.71	−1.8
2	0.17	25	5	2.21	2.19	0.9
3	0.23	28	6	2.84	2.79	1.76
4	0.17	18	5.5	3.2	3.1	3.1
5	0.21	24	7.2	4.1	4	2.4
6	0.14	16	6.8	2.9	2.8	3.4
7	0.23	22	4.5	4.5	4.4	2.2

**Table 10:** Validation test results for the response coating hardness.

Exp. No	Current density (A/cm^2^)	Oxidation time (min)	Inter electrode distance (cm)	Coating hardness (HV_0.5_)		Error (%)
				Actual	Predicted	
1	0.14	15	7	1351	1348	0.22
2	0.17	25	5	1392	1401	−0.64
3	0.23	28	6	1291	1302	−0.84
4	0.17	18	5.5	1285	1290	0.4
5	0.21	24	7.2	1380	1375	0.4
6	0.14	16	6.8	1150	1160	0.9
7	0.23	22	4.5	1420	1415	0.4

**Fig. 7:**
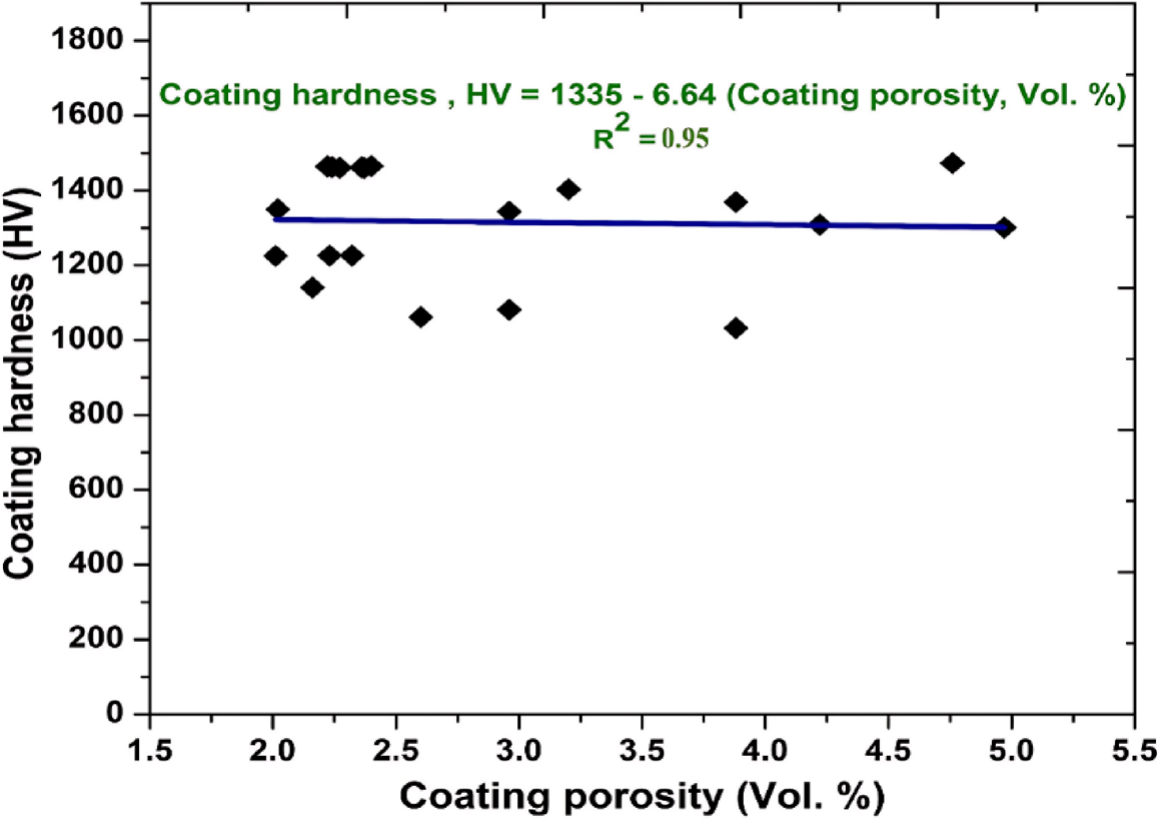
Correlation between porosity and hardness.

Along with the design matrix experiments, independent validation experiments were done with combinations of parameters that were not in the CCD design. As can be seen in the results (Tables 9 and 10), both prediction errors of porosity and hardness are less than 2% indicating a high level of agreement between the experimentally determined and predicted values. These validation findings support the fact that the model created is not restricted to the interpolation in the design space but can be successfully used to predict the responses in the specified parameter space. This once again justifies the strength and realistic practicability of the RSM-based model.

Phase estimation was carried out using a semi-quantitative reference intensity ratio (RIR) approach based on The International Centre for Diffraction Data (ICDD) data, comparing characteristic peaks of γ-Al₂O₃ and α-Al₂O₃, with relative intensities normalized by their respective RIR values. The results show a clear trend with processing conditions. At low current density (0.12 A/cm²), the coating contains roughly γ-phase of around 25% and α-phase of around 25%. At the optimized condition (0.19 A/cm²), γ-phase increases to about 55% with α around 55%, while at higher current (0.25 A/cm²), α-phase drops below 5% and γ becomes dominant (~ 70%), likely due to rapid quenching effects. A similar trend with treatment time is also observed, where γ-phase content increases with duration^29^. Further, these phase variations correlate well with the measured properties, showing improved hardness and reduced porosity under optimized conditions, without any noticeable peak shift or scaling inconsistencies in the XRD data.

### Relationship among porosity and hardness of the coated surface

Table 5 presents the porosity and hardness values obtained by experimentation are illustrated on a line graph (Figure 7). The data points are plotted and linked by a line of best fit, with the equation for this line also provided.

$$\text{Coating hardness (HV)} = 1335 -6.64 \text{(Coating porosity, vol. }\%)$$

Porosity has an inverse relationship with hardness is confirmed by the negative gradient of the best fit (−6.64). The R^2^ is found to be 0.95, indicating a strong inverse correlation consistent with the expectation that denser coatings exhibit higher hardness. This coefficient offers insight into how well the resultant regression equation fits the data. The equation can be used to estimate the average coating hardness based on a specific amount of coating porosity.

## Discussion

### Current density effect on coating porosity and hardness

**Fig. 8:**
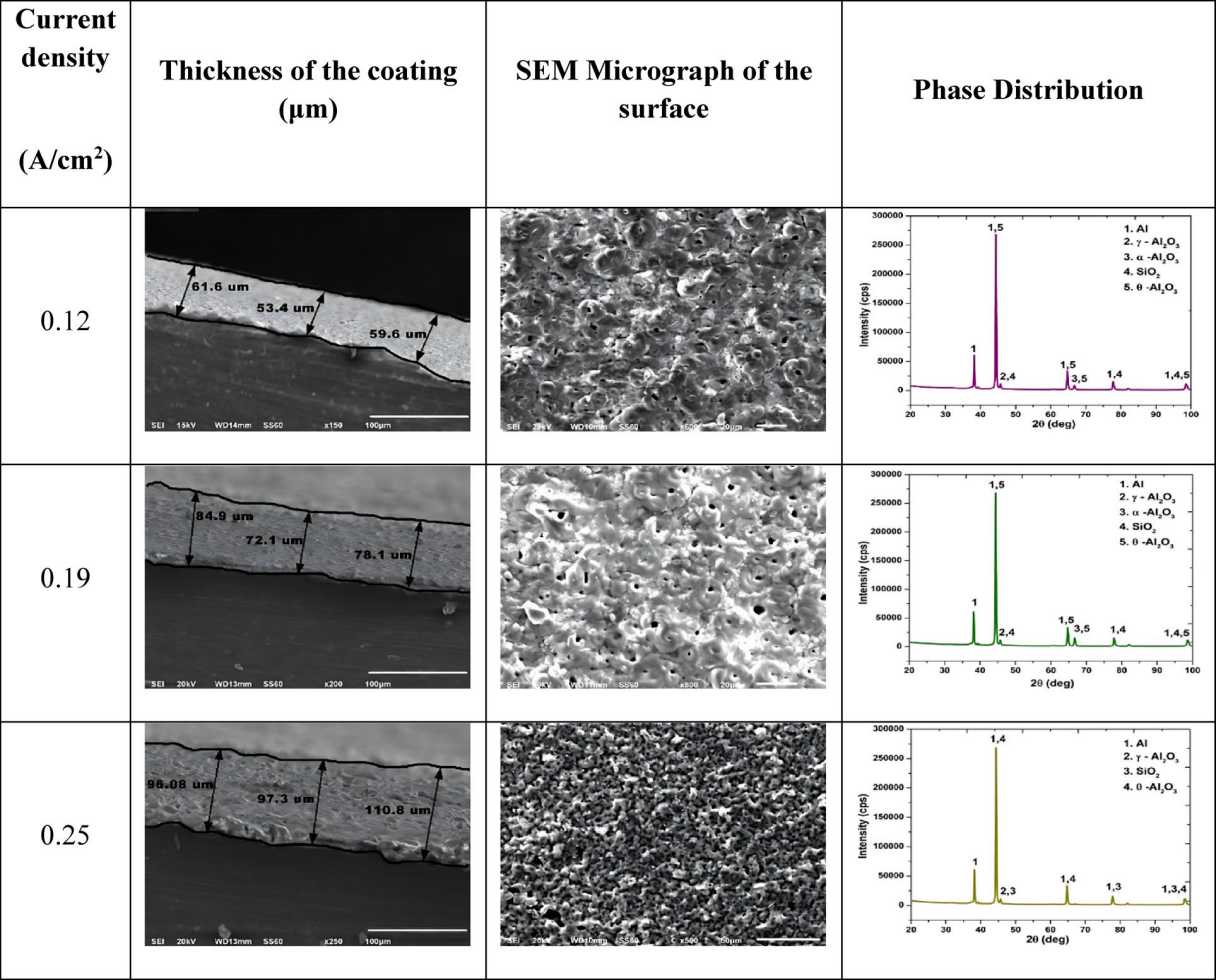
SEM Micrograph of cross section showing the thickness and the top surface and the distribution of phases at various current densities.

The micrograph of the coated specimens is displayed in Fig. 8 and shows the thickness and distribution of phases at different current densities. The sample surface is marked by a great number of holes of different sizes, and the surface can be compared to a volcanic structure. The volcano on the coated surface is formed as a result of spraying the aluminum matrix into surface and its oxide layer via the discharge channels at high pressure and temperature and then solidifying the electrolytic quenching action closes the discharge channels during the MAO process^12,30^. The holes are mainly formed during the overflow of gas through the fractured surface that was formed during the reaction process. The volcanic nature of the surface is attributed to the spraying of the melted oxide layer that is found on the inside. Also, the alumina layer phase transformation, meaning the conversion of non-crystalline phase to γ- Al_2_O_3_ and γ- Al_2_O_3_ to α- Al_2_O_3_, results in the presence of micro-cracks on the surface of the alumina layer^2,31^.

The volumetric changes of the material causes the stress and crack development in the process. Figure 8 also shows the XRD of MAO covered specimens that were exposed to different levels of current density. These stages are α-Al_2_O_3_, θ-Al_2_O_3_, γ-Al_2_O_3_ and SiO_2_. It is worth noting that α and γ - Al_2_O_3_’s are the most common phases present on the MAO coated surface. The XRD plots show that the intensity of the diffraction peaks of α- Al_2_O_3_ also rises with the rise in the current density between 0.12 and 0.19 A/cm^2^. At 0.25 A/cm², the XRD pattern reveals only γ-Al₂O₃, with no detectable α-phase peaks, which is attributed to rapid quenching suppressing α-phase nucleation despite high discharge temperatures. In line with this, the hardness of the MAO coating varies with the current density, which increases by 1141 HV to 1465 HV with the increase in current density between 0.12 and 0.19 A/cm^2^ due to the increase in the percentage composition of α- Al_2_O_3_. Beyond 0.19 A/cm^2^, a slight rise of hardness is detected with a subsequent decrease in hardness at 0.25 A/cm^2^ because only γ- Al_2_O_3_ is observed, as supported by the XRD analysis^12,32^.

The elevated current density (0.12–0.19 A/cm²) generates high-energy discharges (~ 10⁴ K), favoring metastable γ-Al₂O₃ formation near the substrate through rapid Al oxidation and ejection, with SiO₂ or mullite from the Na₂SiO₃-KOH electrolyte; the weld’s Si or Mg aids nucleation^1,33^. At 0.25 A/cm², i.e. at prolonged high temperatures (> 2000 K), metastable γ-Al₂O₃ forms causing volume contraction. This transforms α at 102 Å unit cell to γ at 100 Å unit cell and promotes cracks or porosity by lowering hardness. During MAO processing, the phase balance between γ-Al₂O₃ and α-Al₂O₃ evolves systematically with current density, as reflected in XRD peak intensities and governed by the interplay between discharge energy and cooling rate. At a lower current density of 0.12 A/cm², the plasma discharges are relatively mild, producing limited localized heating; as a result, the coating is dominated by metastable γ-Al₂O₃, identified by its characteristic peaks (~ 45°), while α-Al₂O₃ peaks (~ 67°) remain weak due to insufficient energy for full transformation. Increasing the current density to 0.19 A/cm² intensifies the discharges, raising local temperatures to levels that favor the transformation of γ to the thermodynamically stable α-phase; this is confirmed by the strengthening of α-Al₂O₃ peaks in XRD and corresponds to improved coating densification and hardness. However, when the current density is further increased to 0.25 A/cm², the process enters a highly energetic regime with very rapid quenching; although high temperatures are still achieved, the extreme cooling rates and unstable discharge behavior hinder sustained α-phase growth and instead promote the re-formation or retention of γ-Al₂O₃. This reversal is evident in XRD as a reduction in α-peak intensity and a relative increase in γ-phase features, accompanied by microstructural defects such as porosity and microcracks. Thus, the observed trend an increase in α-Al₂O₃ up to 0.19 A/cm² followed by a decline at 0.25 A/cm² can be explained by a balance between thermal activation for phase transformation and kinetic constraints imposed by rapid quenching under high-energy discharge conditions, consistent with mechanisms reported in the literature^34–36^.

Semi-quantitative phase analysis was carried out using the RIR method by comparing the integrated peak areas of α -Al₂O₃ (113) at ~ 67° (2θ) and γ -Al₂O₃ (220) at ~ 45° (2θ), normalized with standard RIR values (α-Al₂O₃ = 1.0; γ-Al₂O₃ ≈ 2.5, ICDD data). The results indicate that the α-Al₂O₃ content increased from approximately 25% at 0.12 A/cm² to about 55% at 0.19 A/cm², consistent with the observed rise in hardness (1141–1465 HV) as the harder and more stable α phase became predominant. At 0.25 A/cm², the coating was largely composed of γ-Al₂O₃; however, a reduction in hardness was noted, likely due to the formation of microcracks and the higher γ-phase fraction^37,38^.

**Fig. 9:**
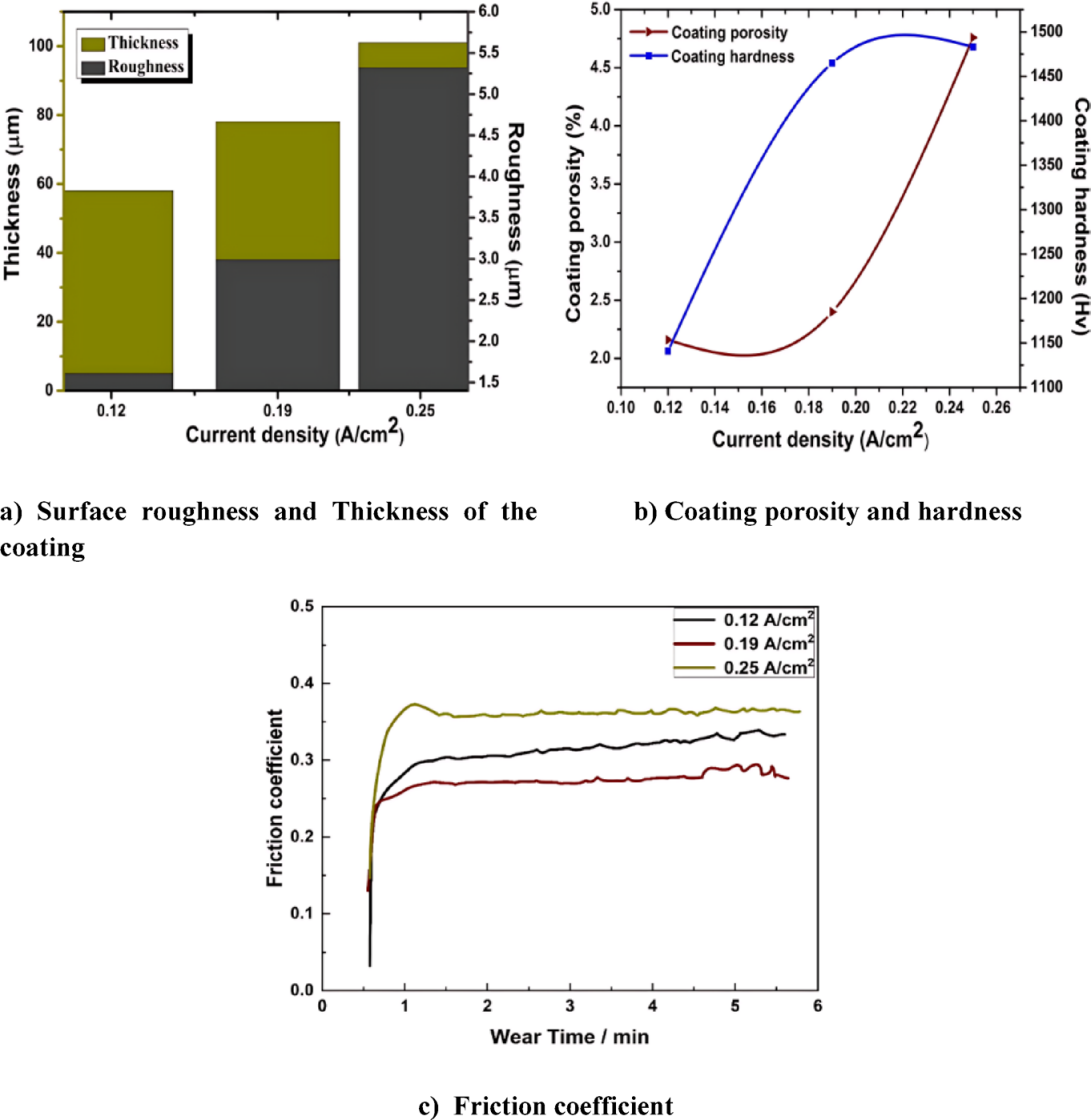
Influence of current density on surface roughness, thickness, porosity, hardness and friction coefficient of the coating.

The effect of I on the coating surface roughness and the thickness is shown in Fig. 9a) and the influence of I on the porosity and hardness of the coating is shown in Fig. 9b). The generation of bubbles through molten oxides released through the channels of discharge formed during the MAO process cause the formation of pores^39^. Increasing the I value reduces pore number while enlarging pore size. In particular, the influence of current density (0.10–0.25 A/cm²) is discussed in relation to earlier work, where the observed peak hardness of 1465 HV exceeds values such as 1200 HV reported at 0.15 A/cm² by^12^, while the reduction in porosity is consistent with findings^40^. An increase in I value (0.12 to 0.19 A/cm^2^) will first cause the thickness of the coatings to increase (58 to 78 μm) but once the I value is increased further (0.19 to 0.25 A/cm^2^) the thickness will increase to 101 μm. The reason is that as the I value rises, the discharges of the spark increase resulting in the development of bigger discharge channels. It is hence clear that the I is a significant factor in the thickness of the coatings. Lower current densities leads to the occurrence of compact coatings whereas higher current densities leads to the occurrence of dense coatings with more micro-cracks. These defects are caused by the melting and solidification of the hard ceramic compounds (Al_2_O_3_) because of the formation of thermal stresses^12,41^.

As illustrated in Fig. 9c) the I value of 0.19 A/cm^2^ has excellent friction and wear resistance with a friction coefficient of approximately 0.23. The ceramic coating friction coefficient curves generated at the different cathodic current densities are seen to be relatively smooth meaning that they have a homogenous texture^17,42^. Nevertheless, the differences in the friction coefficients and curve inclination can be used to evaluate the α- Al_2_O_3_ content qualitatively that gradually increases in the surface to the substrate^43^. The coefficient of friction is 0.27 at I value of 0.12 A/cm^2^. The roughness, hardness, and thickness of the coating following MAO demonstrate that it is wear-resistant than the substrate with a friction coefficient of 0.462. To a certain degree, the wear resistance of the coating should improve as the density of cathodic current increases^44^. But there is an abnormal effect at an I value of 0.25 A/cm^2^ and at that point the coefficient of friction becomes 0.37. This can be attributed to the fact that the surface of coating is rougher as shown by the microstructure of AA6082 ceramic coating and a reduced α-Al phase. Finally, this could be because of the thick coating with a loose layer that has a higher percentage of the γ-Al_2_O_3_ phase, which is indicated by its XRD patterns.

### Inter-electrode distance effect on coating porosity and hardness

**Fig. 10:**
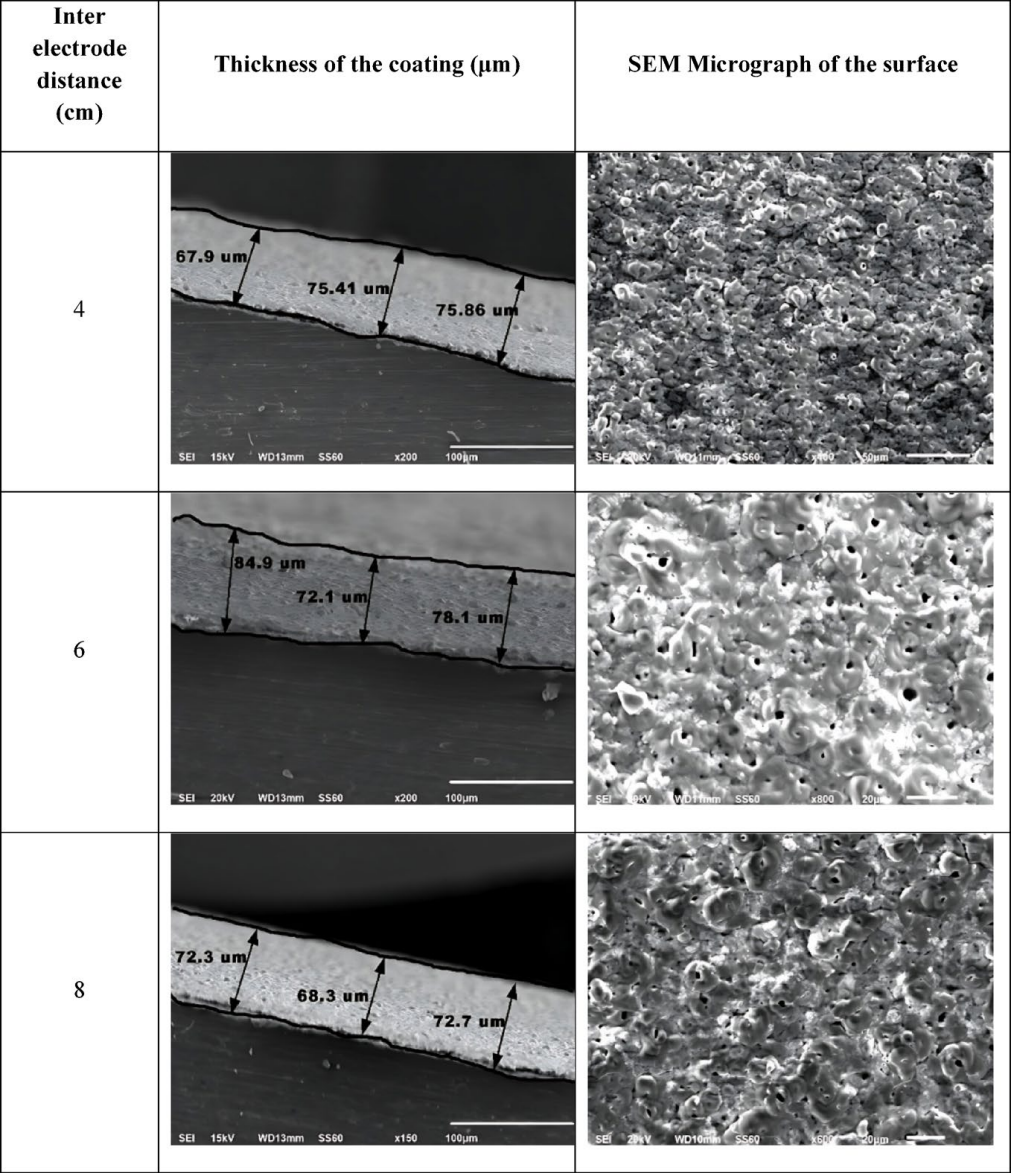
SEM Micrograph of cross section showing the thickness and the top surface at various inter-electrode distances.

The distance between electrodes is also a main parameter that significantly affects the process of MAO, since it determines the intensity of electric fields and distribution of I among electrodes. Inter-electrode distance was also tested by keeping the I value at 0.19 A/cm^2^ and the T value as 20 min in order to determine its effect on the surface. Figure 10 presents the SEM micrograph of coated specimens, showing thickness at different inter-electrode distances. Shorter distances create stronger electric fields and thicker coatings because of more ion movement. But if the distance is too short, it can cause uneven growth and porosity^14,45^.

**Fig. 11:**
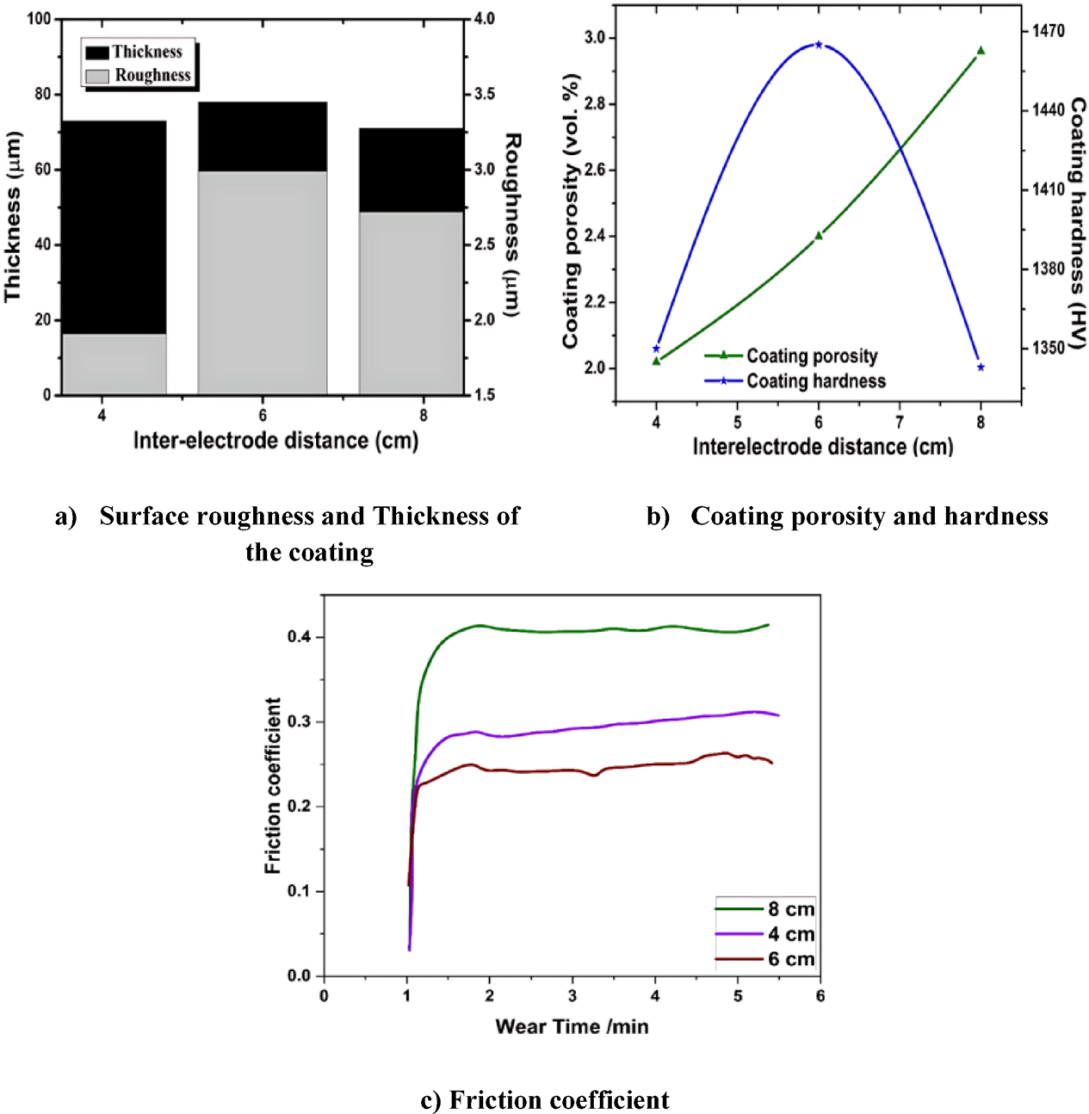
Influence of inter electrode distance on surface roughness, thickness, porosity, hardness and friction coefficient of the coating.

The influence of D value on coating thickness and roughness is shown in Fig. 11a, while Fig. 11b illustrates its effect on coating porosity and hardness. At first, shorter distances reduce porosity and increase hardness. But if the distance is too short, it can have the opposite effect. The effect of D on phase composition is less predictable. The effect of D value of (4–8 cm) was found to be comparatively minor, aligning with observations by^14,46^. Electric field and temperature affect transformation less than I and T. Figure 11c, shows friction coefficient versus inter-electrode distance. Friction usually decreases with shorter distances because of increased hardness and less porosity. But if the distance is too short, friction may increase due to uneven coating and roughness^47^. Variations in D value resulted in minor changes in coating porosity and hardness, indicating that D does not significantly affect pore volume percentage or hardness of MAO coated aluminum alloy joints. This is supported by ANOVA test results, where the Fisher’s ratio for D is lower than that for I and T.

### Oxidation time effect on coating porosity and hardness

**Fig. 12:**
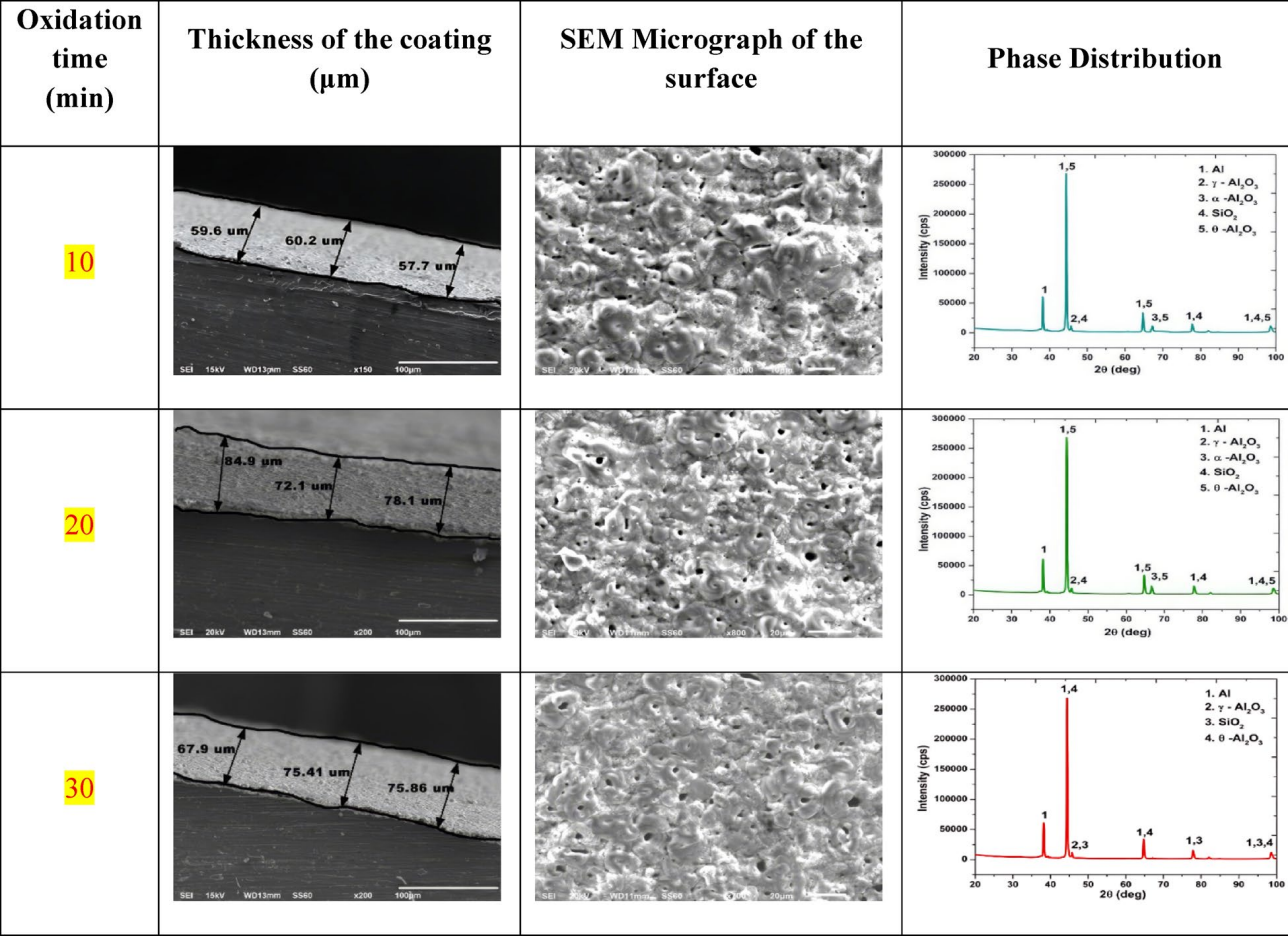
SEM Micrograph of cross section showing the thickness and the top surface showing the distribution of phases at various oxidation times.

**Fig. 13:**
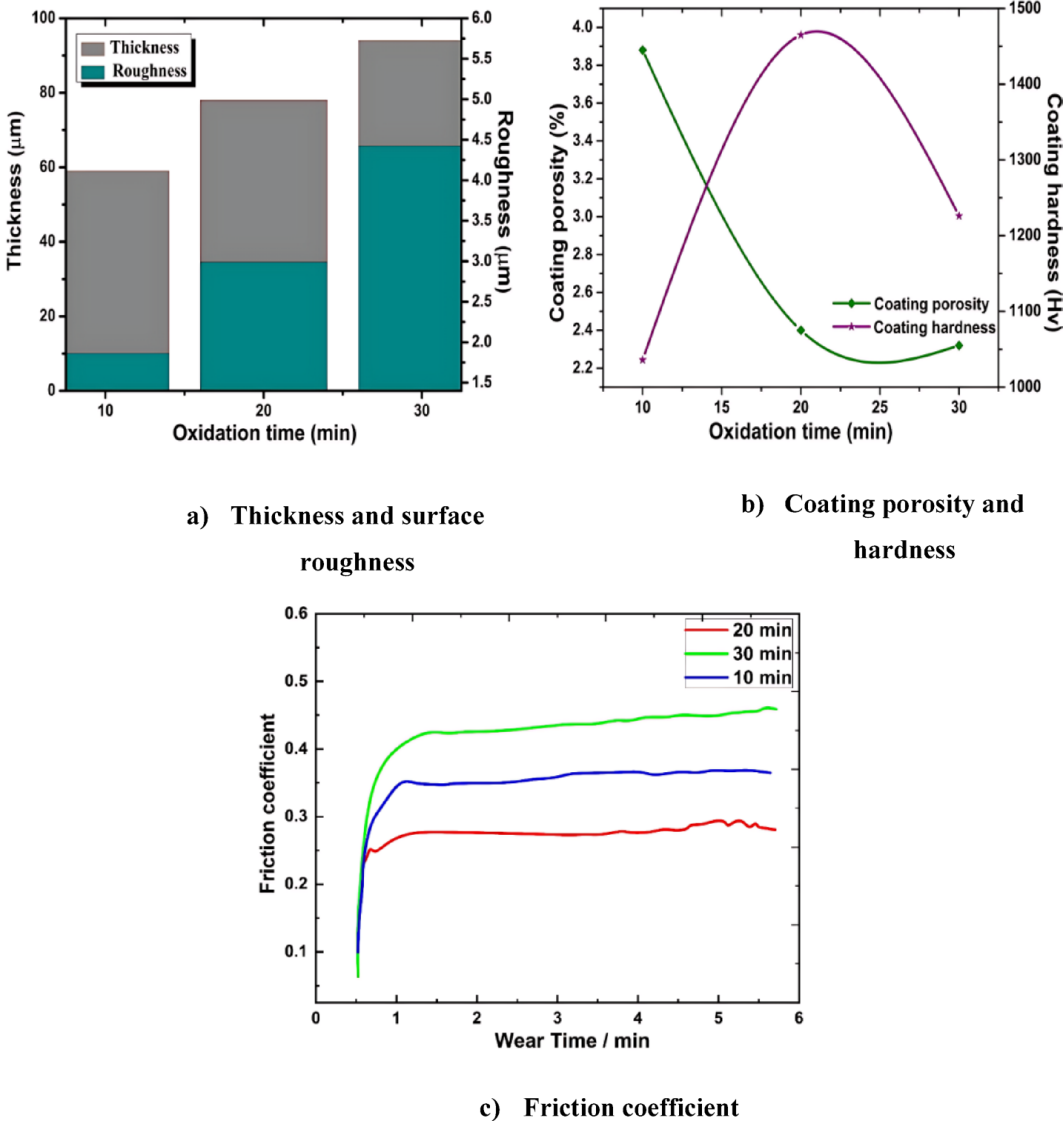
Effect of oxidation time on surface roughness, thickness, porosity, hardness and friction coefficient of the coating.

Figure 12 shows micrograph of cross section showing the thickness and the top surface showing the distribution of phases at various oxidation times with help of SEM and XRD pattern. Extended oxidation increases coating thickness. Initially, small pores appear, becoming larger but fewer over time. Figure 13a, shows thickness and roughness changes. The microstructure may become denser over time. Brief oxidation (10 min) led to incomplete phase transformation, suggesting longer durations upto 20 min enable converting γ-Al₂O₃ and mullite into α-Al₂O₃. Figure 13b) displays porosity, hardness, and surface effects. Porosity decreases while hardness increases until 20 min before declining. Longer oxidation times reduce porosity and increase hardness, but excessive oxidation may cause coating degradation. The optimized T value of 20 min produced a coating thickness of about 78 μm with superior hardness compared to studies such as^9,48^, where 10 min yielded lower hardness (1040 HV). Figure 12 also illustrates the XRD patterns at different oxidation durations. Longer oxidation upto 20 min yields more thermodynamically stable α-Al₂O₃ phase. Figure 13a-c highlights the influence of T on porosity, surface roughness, hardness, friction coefficient and thickness of MAO coated aluminium alloys joint. The coefficient initially decreases with T value due to enhanced hardness but may increase with excessive oxidation. Effect of T on coating porosity and hardness using I value of 0.19 A/cm² and D value of 6 cm, T value of (10–30 min) effects were studied. At shorter times, molten oxides contact cooler electrolyte^30,49^. XRD analysis confirms increased heat and α-Al₂O₃ enhance hardness^9,12,50^. Extended time increases thickness but reduces hardness due to heat effects. Longer oxidation times make thicker MAO coatings. These coatings have better wear resistance because they have denser α-Al₂O₃ layers. The results are consistent with^20,32,51^. Longer oxidation also lowers the friction coefficient to 0.23 compared to 0.27 for shorter times, which helps with lubrication. A load of 330 g, frequency of 15 Hz, duration of 5 min with a 4 mm steel ball were chosen in accordance with ASTM G99 to enable reliable comparison of relative wear performance. The optimized coating exhibited a coefficient of friction of 0.23 compared with 0.46 for the untreated substrate. These conditions are representative of mild sliding environments encountered in certain aerospace applications, corresponding approximately to localized or high magnitude compressive stresses in the range of 0.3–0.5 MPa and vibration frequencies of 10–20 Hz under tribocorrosion scenarios. SEM images show that longer oxidation makes smoother surfaces, which reduces friction. A uniform coating reduces local wear and makes the coating last longer^52^.

### Interaction effect on coating porosity and hardness

The interaction between I and T shows a pronounced influence on coating hardness, as reflected by the relatively high interaction coefficient (−18.25). This behavior can be due to the collective effect of intensified plasma activity and extended oxidation duration during the MAO process. Higher I value promotes the development of larger energetic discharge pathways, which increases localized melting and material ejection at the coating surface. When this is accompanied by a longer treatment time, the repeated discharge events allow the molten regions to gradually transform and densify, promoting the transition from metastable γ-Al₂O₃ to the more stable and harder α-Al₂O₃ phase. I-T interaction drives progression from Stage 1 (amorphous) to Stage 2 (sparking, compact γ-layer) optimally, while excess leads to Stage 3 where arcing occurs; D subtly tunes field for ion flux/thickness. The porosity-hardness inverse (slope − 6.64 HV/vol%) stems from gas trapped melt channels, validated by RSM contours. As illustrated in the microstructural and phase analysis (Figs. 8 and 13), this synergistic condition results in a denser ceramic layer and leads to the maximum hardness value observed in the study (1459.36 HV). In contrast, combinations involving low current density and shorter oxidation times provide insufficient energy input, limiting plasma intensity and restricting phase transformation, which results in coatings dominated by porous γ-Al₂O₃ and consequently lower hardness values.

In comparison, the effect of the I-T interaction on porosity is less pronounced, as indicated by the much smaller interaction coefficient (−9.9 × 10⁻^2^). The overall pore volume is primarily governed by the quadratic effects of I and D, where excessive current density can lead to discharge channel coalescence and the formation of larger pores. Interactions involving inter-electrode distance (I×D and D×T) influence hardness mainly through their role in controlling the uniformity of the electric field within the electrolyte. When the electrode spacing is too small under high values of I, discharge activity becomes uneven and localized, often generating microcracks that reduce coating hardness, as observed in the surface morphology (Figs. 10 and 11). The three-dimensional response plots in Fig. 4, help clarify these relationships and link the statistical analysis to the observed microstructures.

The interaction between current density and treatment time plays a decisive role in coating development, with a highly significant effect on hardness (IT = − 18.25, *p* < 0.0001). Increasing the current density (around 0.19 A/cm²) intensifies the discharge activity, generating high-energy plasma conditions that promote localized melting and material ejection through discharge channels, producing characteristic crater-like surface features. When combined with longer treatment durations (20–28 min), these repeated discharge events support the gradual transformation of metastable γ-Al₂O₃ into the more stable α-Al₂O₃ phase, as confirmed by the strengthening of α-phase peaks in XRD. This phase evolution contributes to volume contraction and pore closure, resulting in reduced porosity (as low as ~ 2 vol%) and enhanced hardness, reaching values close to 1459.36 HV. However, at excessively high current or prolonged duration, the process shifts toward unstable arcing, leading to larger discharge events, thermal stresses, and the formation of microcracks. These defects can trap gases and increase porosity, ultimately reducing hardness^53^. The influence of inter-electrode distance appears comparatively minor, mainly affecting field distribution and coating uniformity. Overall, the combined experimental observations from SEM, XRD, and statistical analysis (R² ≈ 0.9999) confirm a strong correlation between processing conditions, microstructural evolution, and coating performance, consistent with trends reported for similar silicate-based MAO systems^54^.

Therefore, this study on RSM optimization to MAO treatment of CMT-welded AA6082 fusion zones, demonstrates that I is the most influential parameter (F = 11970, *p* < 0.0001) in promoting α-Al₂O₃ formation resulting in very low porosity (2.07 vol%) and high hardness (1459.36 HV). The study further shows that the reduced dilution associated with CMT welding favors α-phase development, with XRD peak intensity correlating strongly with hardness and inversely with porosity (R² = 0.95). The developed quadratic RSM models (R² > 0.99) capture the link between processing parameters and phase evolution, indicating that current densities above 0.19 A/cm² accelerate the γ to α transformation but may also introduce microcracking.

## Conclusions

1. Empirical relationships were developed to estimate the surface characteristics like coating hardness and coating porosity of the CMT welded AA6082 alloy joints incorporating important MAO coating parameters. These relationships can be effectively used to estimate characteristics of the coating at 99% confidence level.
2. Better coating characteristics were achieved under three different combinations of MAO coating parameters. Notably, the optimal coating features, characterized by a minimal porosity of 2.07 vol % and a peak hardness of 1459.36 HV, were achieved with a current density of 0.19 A/cm^2^, oxidation time of 20 min and an inter-electrode distance of 6 cm.
3. Of the three process parameters examined, the current density exerted the most significant impact on the surface properties of the MAO coated specimen, with oxidation time and inter electrode distance following in influence, as indicated by the F ratio.
4. Although the RSM-based optimization of the MAO process produced coatings with low porosity (2.07 vol%) and high hardness (1459.36 HV) on CMT-welded AA6082 joints, the current findings are limited to laboratory-scale specimens (20 × 20 × 3 mm) and focus primarily on the weld zone, without assessing long-term corrosion or fatigue performance under cyclic loading or realistic automotive service conditions; therefore, future work should address scale-up to full-size industrial components, evaluate coating uniformity across weld metal and heat-affected zones, investigate modified or hybrid electrolytes to promote greater α-Al₂O₃ formation and examine the mechanical stability of MAO-treated joints under thermal cycling.

## Data Availability

All data generated or analysed during this study are included in this published article.
